# Supplementary material for: Lateral septum adenosine A2A receptors control stress-induced depressive-like behaviors via signaling to the hypothalamus and habenula
Source: Nat Commun. 2023 Apr 5;14:1880. doi: 10.1038/s41467-023-37601-x (PMC10076302; doi:10.1038/s41467-023-37601-x)
Supplement: Supplementary file 1 — Supplementary Information [file 41467_2023_37601_MOESM1_ESM.pdf]

Supplementary data

Supplementary Table 1

Up-regulation of $A_{2A}R$ in CNS diseases

Position	Associated CNS Disorder	Resourse	Detection methods	Referneces
Hippocampal synapses	Alzheimer's disease (AD)	3xTg AD mice	radioligand binding	Silva et al. 2018 NbDis
		AD patients	Western blot analysis	Temido-Ferreira et al. 2020 Mol Psych
		Aβ-injected mice	radioligand binding	Gonçalves et al., 2019 NbDis
		APP-PS1 mice	radioligand binding	Viana da Silva et al. 2016 Mat Comm
	Aging	aged rats	radioligand binding / Western blot	Rebola et al., 2003 JNPhys
		aged rats	Western blot	Canas et al., 2009 NbAg
	Diabetic neuropathy	NONcNZO10/LtJ mice	radioligand binding	Duarte et al., 2012 Plos1
	Epilepsy	kainate-injected mice	radioligand binding	Canas et al., 2018 eNeuro
		kainate-injected mice	radioligand binding	Augusto et al., 2021 NbDis
		kainate-injected rats	radioligand binding	Cognato et al., 2010 JNC
	Depression	helpless mice	radioligand binding	Machado et al., 2018 Mol Nb
		CUS-stressed mice	radioligand binding	Kaster et al., 2015 PNAS
		restraint stress rats	radioligand binding	Cunha et al., 2006 Neuroscience
	Angelman	Ube3a mice	radioligand binding	Moreira de Sá et al., 2020 NbDis
Cerebrocortical synapses	Attention Deficit Hyperactivity Disorder	SHR rats	Western blot	Pandolfo et al., 2013 EurNPsychPharm
	Epilepsy	kainate-injected rats	radioligand binding/ Western blot	Rebola et al., 2005 Epilepsia
		Febrile seizures rats	radioligand binding	Crespo et al., 2018, EpBeh
	Rasmussen's encephalitis (RE)	RE patients	Western blot	He et al., 2020 Brain Pathol
Cortico-striatal synapses	Parkinson's disease (PD)	6-OHDA-injected rats	radioligand binding	Carmo et al., 2019, BJP
	Huntington's disease (HD)	R6/2 mice	radioligand binding	Li et al., 2015, NdDis

	Restless Leg Syndrome	BID rats	FACS-based synaptometry	Rodrigues et al., 2022, Biomol
Cerebellar synapses	Machado-Joseph disease (MJD)	MJD transgenic mice	radioligand binding/ Mrna analysis	Gonçalves et al., 2017, AnnNeu

CUS: chronic mild unpredictable stressed; Ube3a: ubiquitin protein ligase E3A; SHR: spontaneously hypertensive; BID: Brain iron deficiency.

Supplementary figures and figure legends

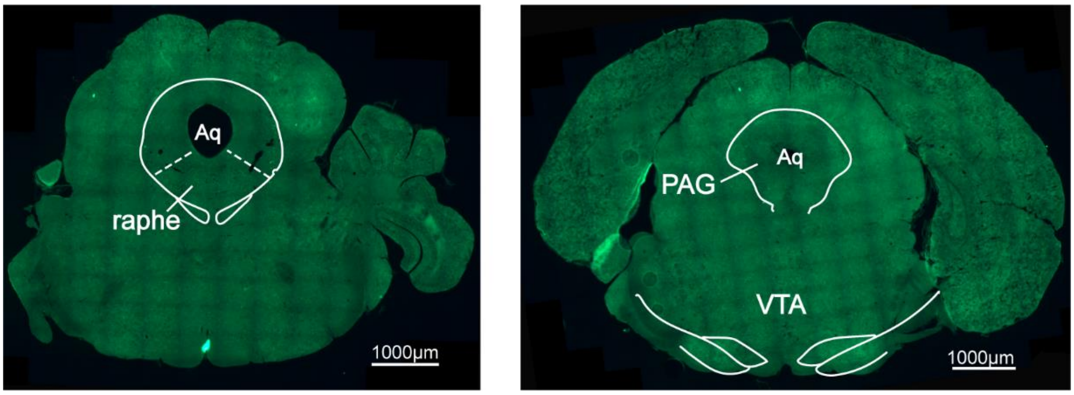

Figure S1. No positive-EYFP signal was found in raphe, periaqueductal gray (PAG) and ventral tegmental area (VTA) .

Confocal images of coronal sections showing no positive-EYFP signal was found in raphe (left), PAG and VTA (right) of A_{2A}R-Cre mice three weeks after receiving AAV2/9-syn-DIO-EYFP injection into the LS. Aq, aqueduct. Scale bar: 1000 μm.

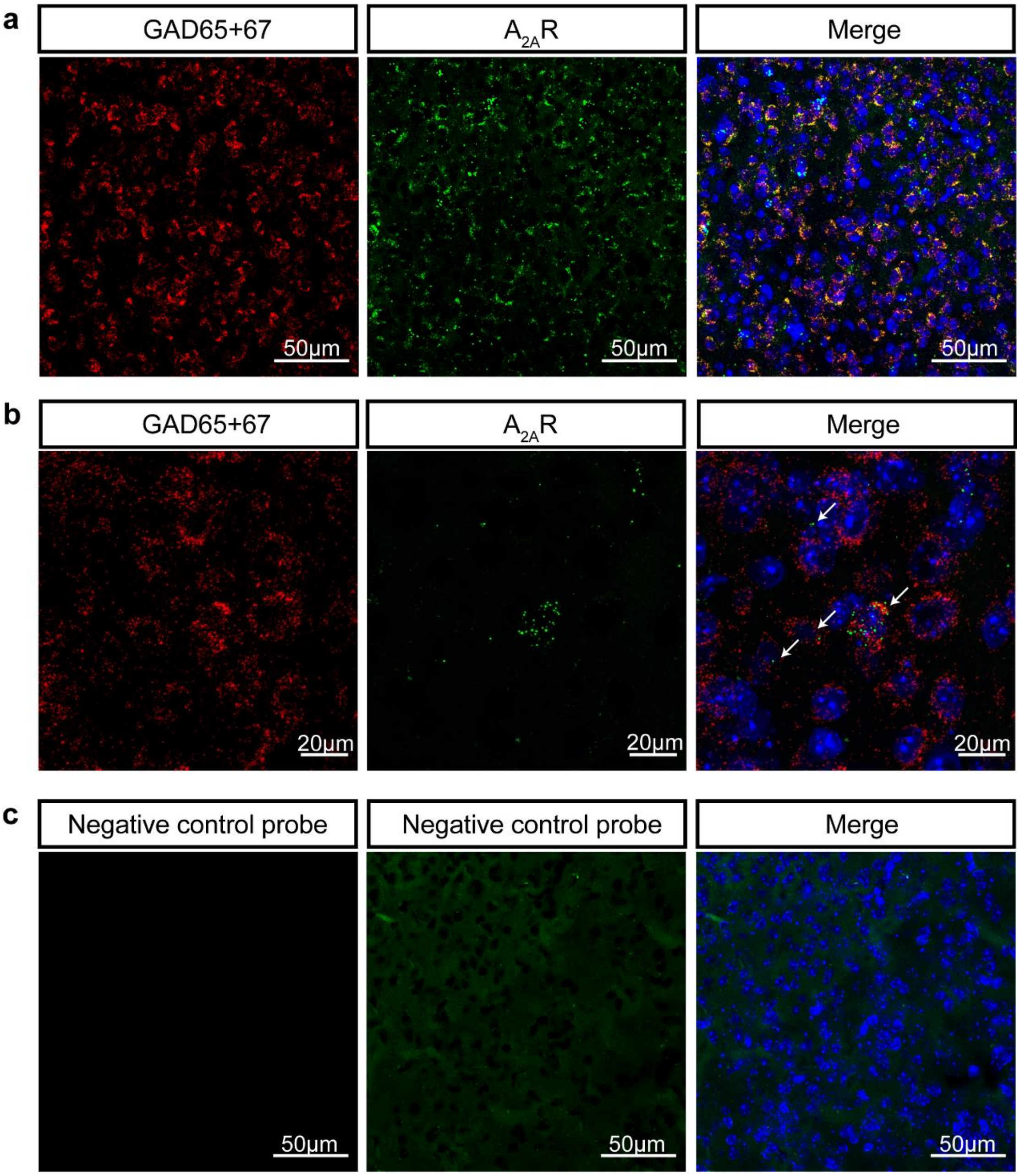

Figure S2. $A_{2A}R$ are expressed essentially in lateral septum (LS)-GABAergic neurons.

(a, b) Representative images of RNAscope *in situ* hybridization using probe against $A_{2A}R$ (green) and GAD65+67 (red) at 20× (A) and 63× (B) magnification in LS of C57BL/6J mice. (c) Representative images using negative control probe (20×). Nuclei are stained with DAPI in blue. Scale bar: 50/20 μm.

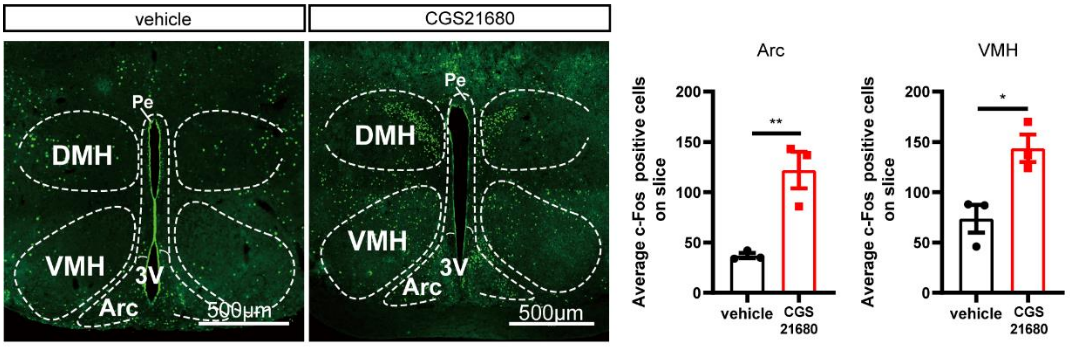

Figure S3. $A_{2A}R$ agonist CGS21680 injection into the lateral septum (LS) increases c-Fos expression in the ventromedial hypothalamic (VMH) and arcuate hypothalamic (Arc)

Representative immunofluorescence images and average bar graphs illustrating the increased expression of c-Fos in the Arc (n=3 mice/group, two-sided Unpaired t test, p=0.0096, t(4)=4.656) and VMH (n=3 mice/group, two-sided Welch's t test, p=0.0228, t(4)=3.596) after focal microinjection of the $A_{2A}R$ agonist CGS21680 into the LS. DMH, dorsomedial hypothalamus; Pe, periventricular hypothalamic nucleus; 3V, 3rd ventricle. Scale bars: 500 μm. Data were shown as mean ± SEM. *$p < 0.05$, **$p < 0.01$. Source data are provided as a Source Data file.

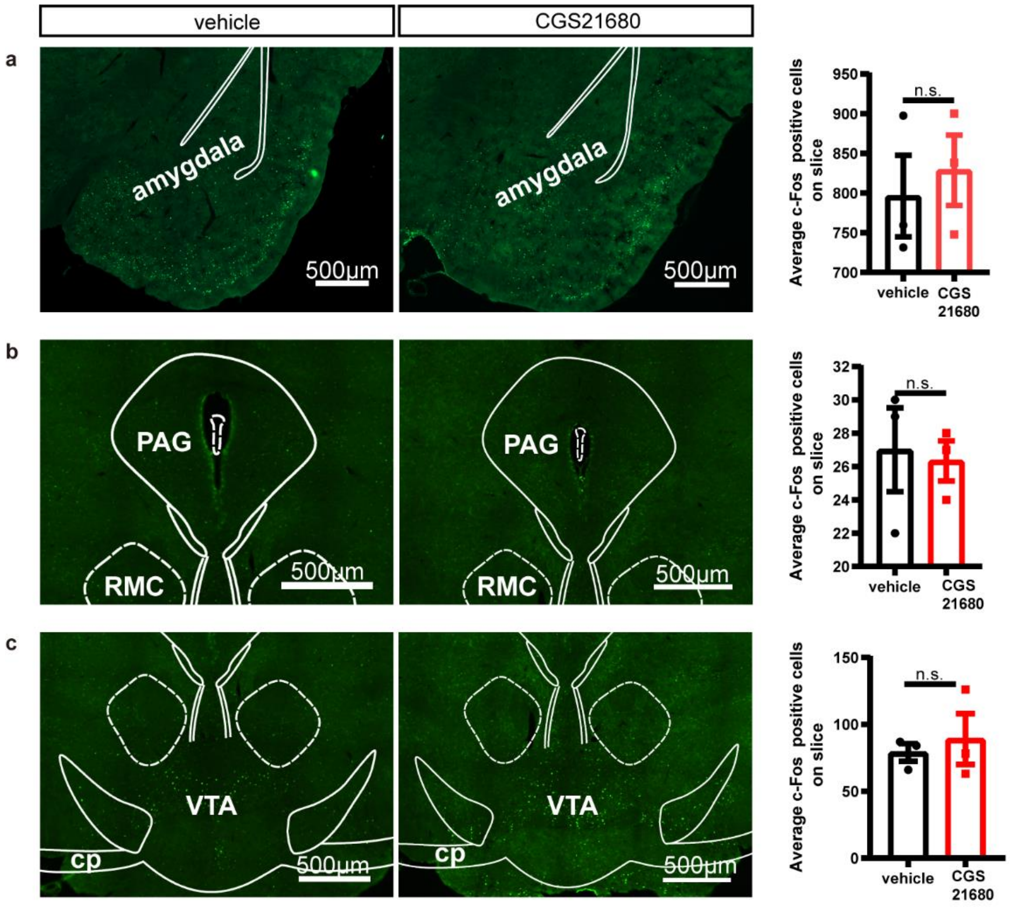

Figure S4. $A_{2A}R$ agonist CGS21680 injection into the lateral septum (LS) do not change c-Fos expression in the amygdala, periaqueductal gray (PAG) and ventral tegmental area (VTA).

Representative immunofluorescence images and average bar graph illustrating the similar expression of c-Fos in amygdala (n=3 mice/group, two-sided Unpaired t test, p=0.6561, t(4)=0.4803) (a), PAG (n=3 mice/group, two-sided Unpaired t test, p=0.8228, t(4)=0.2390) (b) and VTA (n=3 mice/group, two-sided Unpaired t test, p=0.6449, t(4)=0.4975) (c) after focal microinjection of the $A_{2A}R$ agonist CGS21680 into LS. Data are mean ± SEM. RMC: red nucleus, magnocellular part; cp: cerebral peduncle. Data were shown as mean ± SEM, n.s., no significant difference. Source data are provided as a Source Data file.

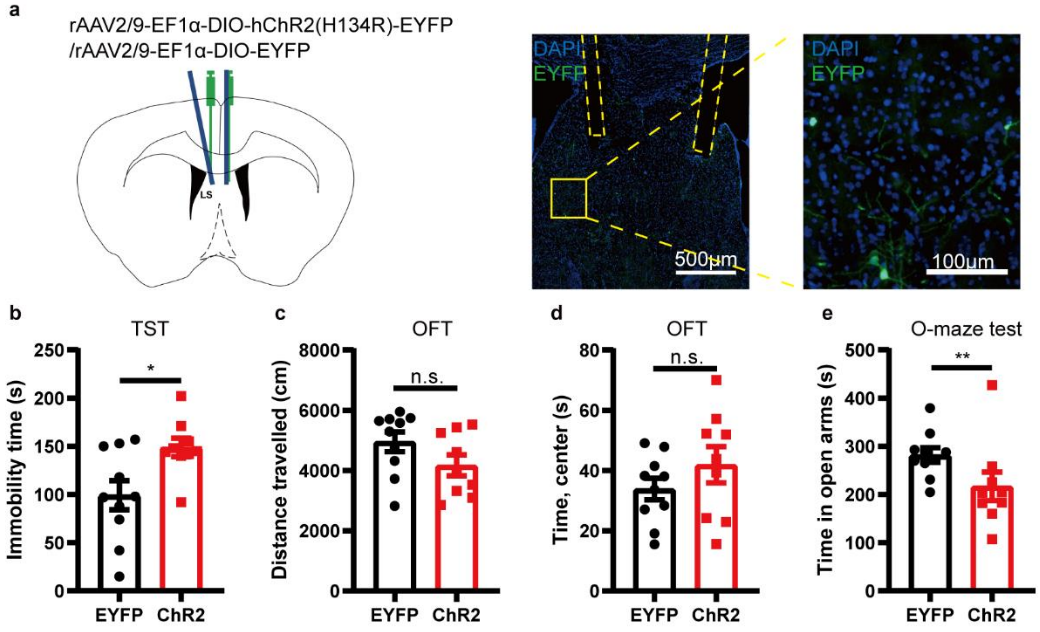

Figure S5. Optogenetic activation of the activity of $A_{2A}R^+$ neurons in the lateral septum (LS) of female A_{2A}R-Cre mice enhances depressive-like phenotype

(a) Left: Schematic illustration of the location of virus injection and optic fibers implantation in female A_{2A}R-Cre mice. Right: A representative fluorescent image showing the ChR2-positive neurons (green) and the localization of the optic fibers. Nuclei are stained with DAPI in blue. Scale bar: 500/100 µm. (b-e) Optogenetic activation of LS-$A_{2A}R^+$ neurons increased the immobility time in the tail suspension test (TST) (n=10 and 9 mice per group for EYFP and ChR2, respectively, two-sided Unpaired t test, *P*=0.0150, t(17)=2.705) (b) and reduced the time in open arms in the O maze test (n=10 and 9 mice per group for EYFP and ChR2, respectively, two-sided Mann-Whitney test, p=0.0081, U=13.50) (e), without affecting the total distance travelled (n=10 and 9 mice per group for EYFP and ChR2, respectively, two-sided Unpaired t test, *p*=0.1226, t(17)=1.625) (c) or the time in the central area in the open field (OF) test (n=10 and 9 mice per group for EYFP and ChR2, respectively, two-sided

Unpaired t test, p=0.2535, t(17)=1.182) (d). Data were shown as mean ± SEM. *p < 0.05, **p < 0.01; n.s., no significant difference. Source data are provided as a Source Data file.

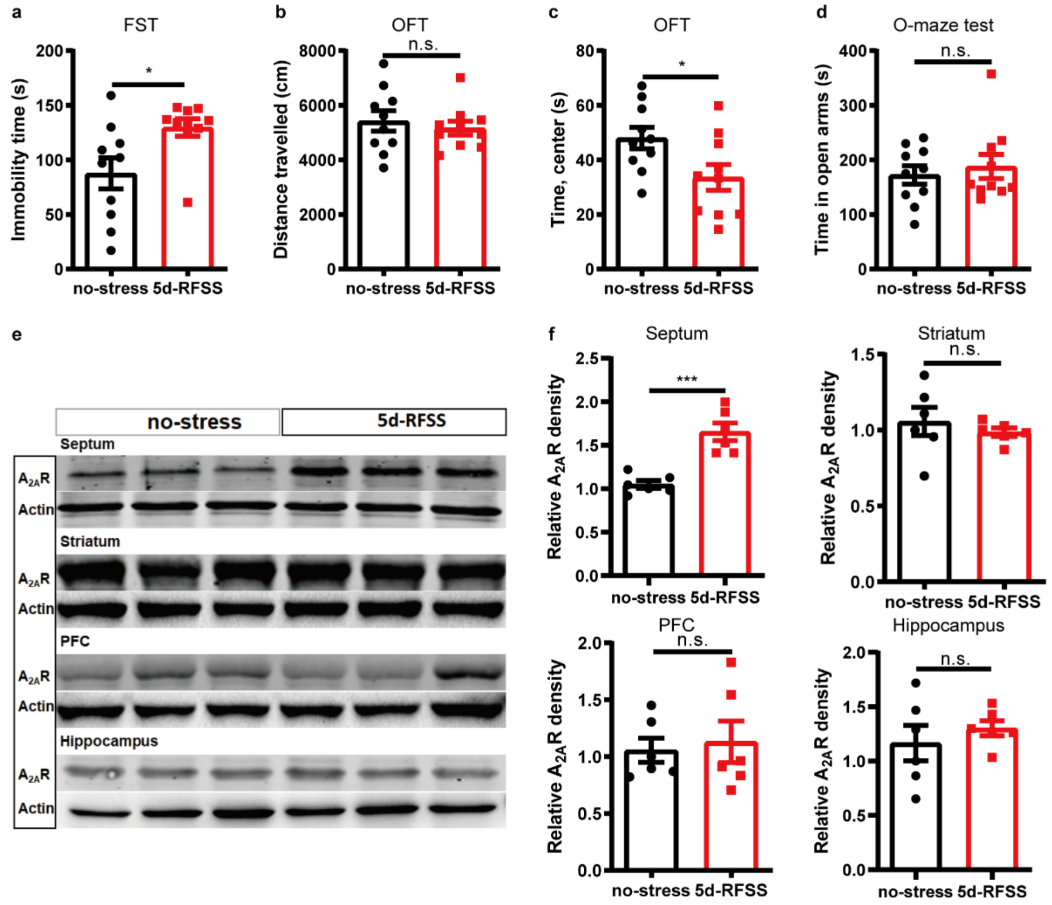

Figure S6. $A_{2A}R$ is selectively upregulated in the lateral septum (LS) in the 5 days repeated forced swim stress (5d-RFSS) model

(a-d) Compared to control mice, 5d-RFSS mice showed increased immobility time in a forced swimming test (FST) (n=10 mice/group, two-sided Mann-Whitney test, *p*=0.0138, U=18) (a) and reduced time in the central area in an open field test (OFT) (n=10 mice/group, two-sided Unpaired t test, *p*=0.0301, t(18)=2.355) (c), without change of the total distance travelled in the OFT (n=10 mice/group, two-sided Unpaired t test, *p*=0.5687, t(18)=0.5806) (b) and time spent in the open arms in an elevated O-maze test (n=10 mice/group, two-sided Mann-Whitney test, *p*=0.7394, U=45) (d). (e, f) Representative Western blot and quantification of $A_{2A}R$ protein levels in the septum (n=6 mice/group, two-sided Unpaired t test, *p*=0.0003, t(10)=5.496), striatum (n=6 mice/group, two-sided Unpaired t test, *p*=0.4878, t(10)=0.7203), prefrontal cortex (PFC) (n=6 mice/group, two-sided Unpaired t test, *p*=0.7341, t(10)=0.3493) and hippocampus (n=6 mice/group, two-sided Unpaired t test, *p*=0.4580,

t(10)=0.7719) of 5d-RFSS and control mice. 5d-RFSS mice displayed a selective upregulation of $A_{2A}R$ in the septum without significant changes in the other three brain regions associated with mood processing. Data were shown as mean ± SEM. *$p < 0.05$, ***$p < 0.001$; n.s., no significant difference. Source data are provided as a Source Data file.
